# Increased Fibrinolysis as a Specific Marker of Poor Outcome After Cardiac Arrest

**DOI:** 10.1097/CCM.0000000000003352

**Published:** 2018-09-14

**Authors:** Nina Buchtele, Christian Schörgenhofer, Alexander O. Spiel, Bernd Jilma, Michael Schwameis

**Affiliations:** 1 Department of Clinical Pharmacology, Medical University of Vienna, Vienna, Austria.; 2 Department of Emergency Medicine, Medical University of Vienna, Vienna, Austria.

**Keywords:** cardiac arrest, early prediction, fibrinolysis, point-of-care, thrombelastometry, outcome

## Abstract

**Objectives:**

Recent data suggest that early increased fibrinolysis may be associated with unfavorable prognosis in cardiac arrest. The current study aimed to assess whether there is an optimal fibrinolysis cutoff value as determined by thrombelastometry at hospital admission to predict poor outcome in a cohort of adult patients with out-of-hospital cardiac arrest.

**Design:**

Prospective observational cohort study.

**Setting:**

Emergency department of a 2.100-bed tertiary care facility in Vienna, Austria, Europe.

**Patients:**

Patients with out-of-hospital cardiac arrest of presumed cardiac origin, subjected to targeted temperature management, who had achieved return of spontaneous circulation at admission were analyzed.

**Interventions:**

None.

**Measurements and Main Results:**

Fibrinolysis was assessed by thrombelastometry at the bedside immediately after hospital admission and is given as maximum lysis (%). The outcome measure was the optimal cutoff for maximum lysis at hospital admission to predict poor outcome (a composite of Cerebral Performance Category 3–5 or death) at day 30, assessed by receiver operating characteristic curve analysis. Seventy-eight patients (61% male, median 59 yr) were included in the study from March 2014 to March 2017. Forty-two patients (54%) had a poor 30-day outcome including 23 nonsurvivors (30%). The maximum lysis cutoff at admission predicting poor 30-day outcome with 100% specificity (95% CI, 90–100%) was greater than or equal to 20%. Tissue-type plasminogen activator antigen levels were likewise elevated in patients with poor neurologic outcome or death 52 ng/mL (interquartile range, 26–79 ng/mL) versus 29 ng/mL (interquartile range, 17–49 ng/mL; *p* = 0.036).

**Conclusions:**

Increased fibrinolysis at admission assessed by thrombelastometry specifically predicts poor outcome in cardiac arrest with presumed cardiac etiology.

## Abstract 2

Supplemental Digital Content is available in the text.

## Background

The process of fibrinolysis is inevitable to regain microvessel patency and restore vital organ perfusion after intravascular clotting. Endothelial tissue-type plasminogen activator (t-PA) ensures clot dissolution by converting plasminogen into plasmin at the site of primary vascular damage averting permanent circulatory compromise and subsequent thrombotic organ failure. In contrast, primary hyperfibrinolysis occurs without preceding intravascular clotting and is associated with poor outcome in several critical conditions including trauma and sepsis (1, 2). In particular in traumatic coagulopathy, fibrinolysis has been investigated intensively and identified as an early and independent predictor of mortality (1, 3).

Although underlying mechanisms likely differ from that in trauma-associated fibrinolysis, which involves tissue injury and significant crystalloid hemodilution (4), previous studies likewise reported an association between the presence of fibrinolysis and unfavorable prognosis in cardiac arrest (5, 6), with the highest fibrinolytic activity found in patients with early death.

Hypoperfusion resulting in release of endothelial t-PA (6) is considered one possible mechanism of fibrinolysis occurring during cardiopulmonary resuscitation (CPR) (7). The importance of hypoxia as a causal factor is best seen in the severe hyperfibrinolysis of young and previously healthy drowning patients where preceding activation of coagulation can be excluded (6). Hence, the extent of fibrinolysis at admission reflects prolonged or insufficient resuscitation efforts and may therefore provide reliable prediction of poor outcome (6).

In this context, previous studies have already identified fibrin degradation products, such as d-dimer, as an early indicator of unfavorable outcome in cardiac arrest (8, 9). D-dimer is a well-known marker of fibrinolysis and can be routinely assessed, but however lacks specificity, which makes the definition of a reliable predictive threshold unlikely.

Yet, thrombelastometry has proven useful for point-of-care detection of increased fibrinolysis in cardiac arrest (6, 7, 10).

The current study aimed to assess whether there is an optimal fibrinolysis cutoff value assessed by thrombelastometry to predict poor outcome in a preselected cohort of successfully resuscitated adult patients with out-of-hospital cardiac arrest.

## METHODS

### Study Population

Eligible for inclusion were adults (≥ 18 yr) with out-of-hospital cardiac arrest of presumed cardiac origin, subjected to targeted temperature management, who had achieved return of spontaneous circulation (ROSC) at admission to the ICU section of the emergency department at the Medical University of Vienna. Exclusion criteria comprised thrombolytic therapy and application of intravascular cooling or extracorporeal bypass device, as both may affect fibrinolytic activity. All patients were treated with therapeutic hypothermia at a target temperature range of 33°C ± 1°C. Patients were cooled using cooling pads (EMCOOLS Flex.Pad, Emcools AG, Pfaffstaetten, Austria) (11) or water-circulating gel-coated pads (Arctic Sun 5000 Temperature Management System; Medivance, Louisville, CO) with or without cold fluids. Target temperature between 32°C and 34°C was maintained for 24 hours after first achievement of less than 34°C. Rewarming was performed at a rate of 0.25–0.5°C per hour. Body temperature was recorded with an esophageal and bladder probe.

Resuscitation-related variables were analyzed and reported according to Utstein recommendations as described previously (12). The primary endpoint was a composite of poor neurologic function or death, defined as a Cerebral Performance Category (CPC) of 3–5 (severe cerebral disability; coma or vegetative state; or brain death, respectively) at day 30 post resuscitation (13). Neurologic function at 30 days was assessed by study fellows through structured face-to-face interview with the patient or by means of structured telephone interview with the patient, the relatives, treating physicians, or nursing home members. Study fellows assessing outcome were all blinded to results obtained by rotational thrombelastometry (ROTEM).

Sustained ROSC was defined as recovery of spontaneous circulation for more than 20 minutes. No- and low-flow intervals were defined as the time from collapse to initiation of CPR and the time from CPR initiation to sustained ROSC, respectively. No- and low-flow intervals were determined through immediate structured telephone interviews with the dispatch center, the emergency physicians, and paramedics at the scene and the bystander who performed the emergency call. The International Society on Thrombosis and Haemostasis disseminated intravascular coagulation (DIC) eight-point score was applied for DIC calculation (14). A score greater than or equal to 5 is compatible with overt DIC (**Supplemental Table 1**, Supplemental Digital Content 1, http://links.lww.com/CCM/D848).

For sample size calculation, we used the methods described by Buderer (15) based on specificity. We anticipated a specificity level of 90% with 95% CI (width of 10%) and a given prevalence of hyperfibrinolysis of 40% based on previously published results (7, 10). The high level of anticipated specificity was estimated based on our own results from drowning patients, where a very high predictive value for death was demonstrated, if signs of hyperfibrinolysis were present (6).

The study was approved by the Ethics Committee of the Medical University of Vienna and carried out in accordance with the Declaration of Helsinki. A waiver was obtained for informed consent at admission, and patients were informed of their study participation on regaining consciousness.

### Laboratory Methods

Blood sampling for thrombelastometry and laboratory studies were performed as soon as vascular access was available. Analysis of whole blood viscoelastic properties was done in 3.8% sodium-citrated whole blood samples using ROTEM (TEM International GmBH, Munich, Germany) as described previously (16). The following ROTEM tests were applied: EXTEM, which tests the extrinsic pathway of coagulation, and APTEM, which corresponds to EXTEM but additionally comprises the antifibrinolytic aprotinin to definitively reveal the presence of increased fibrinolysis. Fibrinolysis is given as maximum lysis (ML) (%), which represents the percentile difference between the highest and lowest clot amplitude (**Supplemental Fig. 1**, Supplemental Digital Content 1, http://links.lww.com/CCM/D848; reference range < 15%, as specified by the manufacturer).

For enzyme-linked immunoassay (ELISA) analysis, blood was collected into tubes containing EDTA or 3.8% citrated plasma. Obtained samples were centrifuged for 10 minutes at 2,000*g*. Plasma was stored at –80°C until being tested. Tissue plasminogen activator antigen (TECHNOZYM t-PA Combi Actibind; Technoclone, Vienna, Austria), plasminogen activator inhibitor (PAI)–1 (TECHNOZYM PAI-1 Actibind; Technoclone) (16), and prothrombin fragments F1 + 2 (EnzygnostF 1 + 2; Siemens, Marburg, Germany) (17) assays were performed using commercially available ELISA kits. The lower limits of detection are 0.01 ng/mL (t-PA antigen), 20 pmol/L (F1 + 2), and 0.49 IU/mL (PAI-1), respectively. All assays were performed according to manufacturer’s instructions.

### Statistical Analysis

Variables are presented as absolute values (*n*), relative frequencies (%), and median (interquartile ranges [IQRs]). Prevalence of increased fibrinolysis and overt DIC is given as a proportion with a 95% CI. Potentially missing data for demographic variables were not imputated. Between-group comparisons were performed using the Mann-Whitney *U* test for continuous variables or the chi-square test/Fisher exact test for nominal variables. We performed exact bivariable logistic regression to estimate the effect on poor neurologic function or death at day 30 of ML and remaining candidate predictors, which were judged to be clinically plausible, including age, no-flow time, low-flow time, pH, lactate, d-dimer levels, sex, CPC prior to cardiac arrest, arrest site, witness status, presence or absence of bystander resuscitation, initial rhythm, epinephrine dose administered during resuscitation, and the rate of sustained ROSC at admission. Results are given as odds ratios/median unbiased estimates with 95% CI and are available in **Supplemental Table 4** (Supplemental Digital Content 1, http://links.lww.com/CCM/D848).

The optimal cutoff for ML to predict poor 30-day outcome was assessed by computing a receiver operating characteristic curve. Specificity and sensitivity were calculated with 95% CI. The Kaplan-Meier method was used to describe survival according to the optimal cutoff for ML.

Generally, a two-sided *p* value of less than 0.05 was considered statistically significant. We used IBM SPSS Statistical Software, Version 22.0 (IBM Corp., Armonk, NY) and Stata Statistical Software: Release 15 (StataCorp., College Station, TX) for statistical analysis and GraphPad Prism Version 7.00 for Windows (GraphPad Software, La Jolla, CA) to draw figures.

## RESULTS

Seventy-eight patients (median age 59 yr; 47–69; 78% male) were included in the study. Results from thrombelastometry at admission and all data for the primary outcome were available. Median core temperature at admission was 35.3°C (34.8–35.8°C). Fibrinolysis exceeding the normal reference value of 15% was present in 36% of patients (28/78; 95% CI, 25–48%). The rate of overt DIC was 4% overall (3/78; 95% CI, 1–11%). In total, 54% of patients (42/78) had a poor 30-day outcome including 23 nonsurvivors (30%). Kaplan-Meier estimates of survival to day 30 according to ML cutoff of greater than or equal to 20% are available with the supplement (**Supplemental Fig. 2**, Supplemental Digital Content 1, http://links.lww.com/CCM/D848).

While nine patients died due to multiple organ failure, in 14 patients a decision to withdraw life-sustaining therapy was made by treating physicians after determination of unfavorable neurologic prognosis. Characteristics of the study patients according to 30-day outcome (CPC) are shown in **Table 1**.

**TABLE 1.**
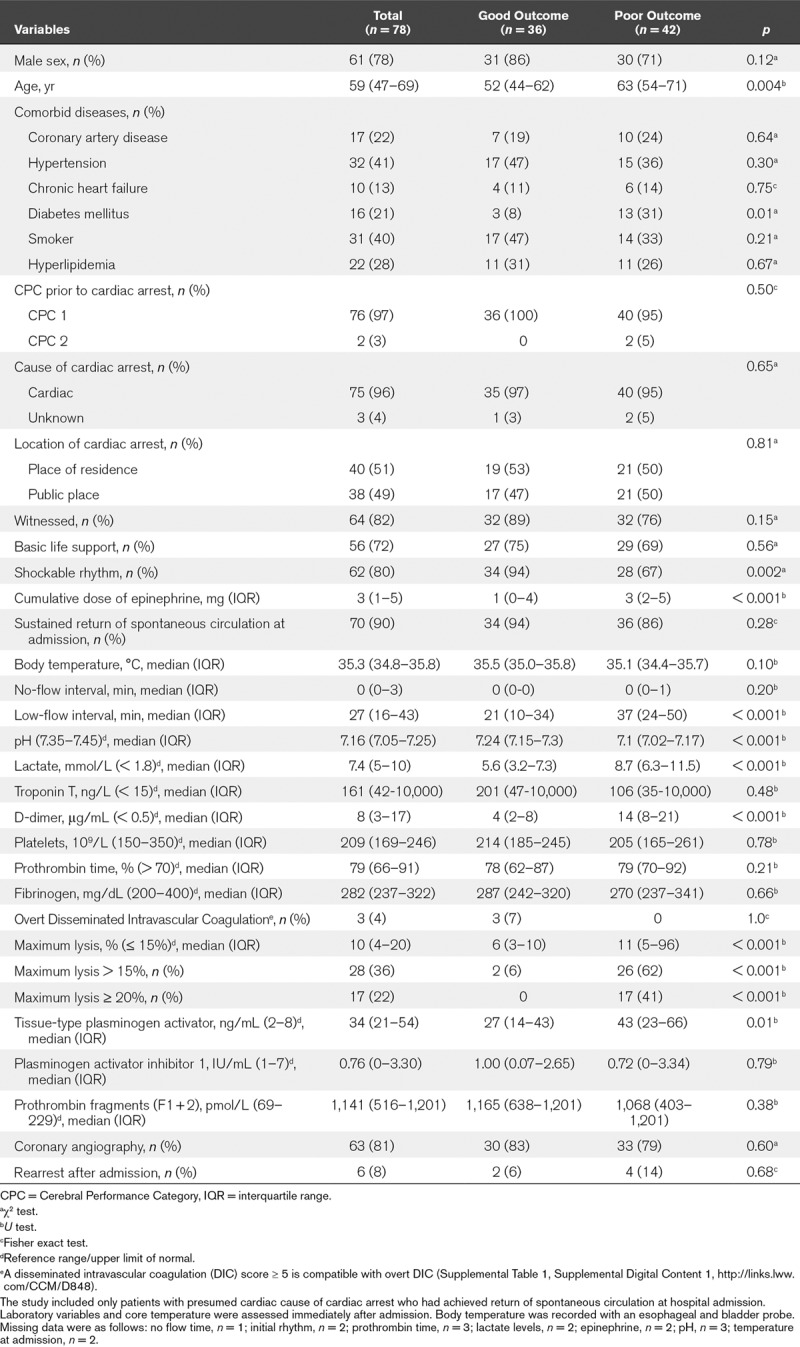
Characteristics of the Study Patients According to 30-Day Outcome

**Figure 1.**
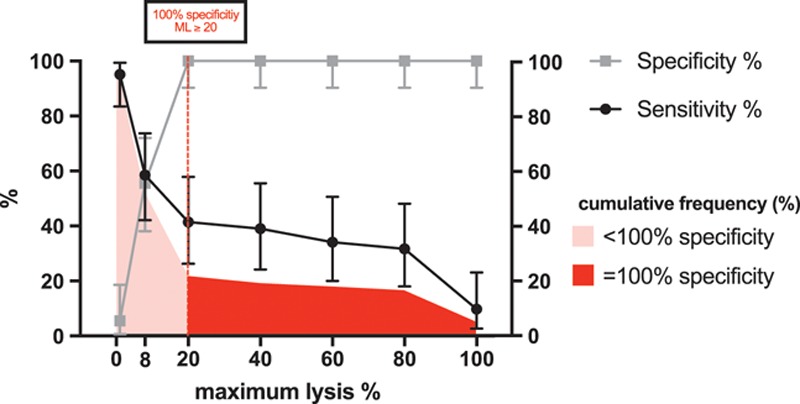
Sensitivity and specificity (%) of increasing fibrinolysis values (maximum lysis [ML], %) to predict poor neurologic function or death at day 30 after admission (*n* = 78). Specificity (*gray squares*), sensitivity (*black dots*, both *left y*-axis), cumulative frequency distribution (%, *light-colored* and *rich-colored red area, right y-axis*), 100% specificity value (*vertical dashed red line*), percentage of patients predicted with 100% specificity to have poor neurologic function or death (*rich-colored red area*). *Error bars* indicate 95% CI. ML equal to or greater than 20% had a 100% specificity for poor neurologic function or death. ML greater than or equal to 20% enables 100% poor outcome prediction in every fifth successfully resuscitated cardiac arrest patient.

The admission ML cutoff predicting poor outcome with 100% specificity (95% CI, 90–100) was greater than or equal to 20% (sensitivity 41%; 95% CI, 26–58) (**Fig. 1**). The corresponding positive and negative predictive values were 100% and 59%, respectively. The receiver operating characteristic curve of ML for prediction of poor 30-day outcome as well as specificity, sensitivity, and cumulative frequency distributions of covariables are given with the supplement (**Supplemental Figs. 3** and **4**, Supplemental Digital Content 1, http://links.lww.com/CCM/D848).

Patients presenting with a ML greater than or equal to 20% (17/78, 22%; 59% male) were older (median 64 yr [IQR, 56–71 yr] vs 56 yr [46–67 yr]; *p* = 0.004) and tended to have a higher frequency of nonshockable rhythm (35% vs 15%; p = 0.09), had on average a 83% longer low-flow time (44 min [35–58 min] vs 24 min [15–36 min]; *p* < 0.001), slightly higher lactate (8.3 mmol/L [6–14 mmol/L] vs 6.9 mmol/L [4–10 mmol/L]; *p* < 0.001), 2.7-fold higher d-dimer (20 μg/mL [10–37 μg/mL] vs 7.4 μg/mL [2.7–14 μg/mL]; *p* < 0.001), and 79% higher t-PA levels (52 ng/mL [26–79 ng/mL] vs 29 ng/mL [17–49 ng/mL]; *p* = 0.036) and tended to receive higher doses of epinephrine (3 mg [2–6 mg] vs 2 mg [1–4 mg]; *p* = 0.06). Median core temperature at admission (35.3°C [34.0–35.4°C] vs 35.3°C [34.8–35.8°C]; *p* = 0.34), platelet counts (215 × 10^9^/L [165–293 × 10^9^/L] vs 205 × 10^9^/L [176–244 × 10^9^/L]; *p* = 0.55), prothrombin times (80% [68–93%] vs 77% [64–90%]; *p* = 0.20), fibrinogen levels (238 mg/dL [167–335 mg/dL] vs 295 mg/dL [246–322 mg/dL]; *p* = 0.08), PAI-1 levels (1.15 IU/mL [0.14–2.87 IU/mL] vs 0.70 IU/mL [0–3.32 IU/mL]; *p* = 0.60), and prothrombin fragments levels (1,201 pmol/L [518–1,201 pmol/L] vs 1,134 pmol/L [513–1,201 pmol/L]; *p* = 0.82) were similar compared with patients without increased fibrinolysis. Patients presenting with a ML greater than or equal to 20% had a median time to death of 6.5 days. The time course of fibrinolysis from admission to rewarming is available in **Supplemental Tables 2 and 3** (Supplemental Digital Content 1, http://links.lww.com/CCM/D848). ML greater than or equal to 20% was an independent predictor of poor neurologic function or death (Supplemental Table 4, Supplemental Digital Content 1, http://links.lww.com/CCM/D848).

## DISCUSSION

The current study prospectively investigated the value of fibrinolysis to predict 30-day outcome early after cardiac arrest. The study was built on previous studies identifying hypoperfusion and hypoxia as triggers of primary fibrinolysis (6, 7). Although prolonged or poor resuscitation efforts may cause hyperfibrinolysis, the prognostic relevance of fibrinolysis occurring during CPR, however, remained unclear. This study specifically tested the hypothesis that increased fibrinolysis in thrombelastometry may predict poor outcome.

The prevalence of increased fibrinolysis (ML > 15%) in our study is in good agreement with previous findings (10). However, we were interested in the optimal cutoff for fibrinolysis to specifically predict poor outcome. The greater than or equal to 20% cutoff value that predicted poor outcome with 100% specificity found in the current study corresponds well to the cutoff for increased fibrinolysis recently observed in healthy volunteers (18).

From previous research, we interpret fibrinolysis as a marker of tissue hypoperfusion due to prolonged resuscitation efforts or poor-quality CPR performance and consecutive accumulation of t-PA (6). This hypothesis is mainly based on data obtained from patients with drowning-related out-of-hospital cardiac arrest (OHCA), who are characterized by severely prolonged no- and low-flow intervals along with massive bleeding at hospital admission (6). Their bleeding phenotype was accompanied by increased t-PA levels and absent clotting signature in thrombelastometry, which were reversed by adding aprotinin in vitro. A subsequent forearm-ischemia model conducted in healthy volunteer confirmed the increase of t-PA levels following interruption of arterial blood flow. Further previous studies likewise reported high plasma fibrinolytic activity in cardiac arrest patients with early death (5) and a good correlation between t-PA levels and markers of hypoperfusion (7). In accordance, patients with ML greater than or equal to 20% had significantly higher t-PA levels at admission. Levels of the t-PA inhibiting protein PAI-1 were comparatively low in patients with and without increased fibrinolysis, which is in agreement with current literature (19). This relates to the lack of a storage compartment for PAI-1—in contrast to t-PA stored in endothelial cells—which forbids a readily available release upon hypoxic conditions. Upon acute t-PA release, PAI-1 is consumed (20, 21), and therefore it seems plausible that its activity is low in the very early phase after arrest. PAI-1 levels have been found to likewise increase only within 6 hours after acute hypoxia, contributing to the fibrinolytic shutdown in the subacute phase of the postcardiac arrest syndrome (5, 22).

Low-flow times and lactate levels in the current study were significantly higher in patients with a ML greater than or equal to 20%, which supports the above findings. Likewise, the rate of nonshockable rhythm tended to be higher, which may suggest longer preceding no-flow intervals before initiation of CPR in these patients. No-flow times did not differ significantly between groups, but are, however, usually only estimates with low reliability.

We suggest that fibrinolysis is mainly triggered by hypoperfusion which could also explain the low negative predictive value of ML found in the current study. It is conceivable that patients with prolonged no-flow times but high-quality CPR performance—and thus, hypothetically, rapid clearance of t-PA—suffer poor neurologic recovery despite lack of fibrinolysis at admission. The quality of CPR performance is, however, difficult to assess routinely.

An alternative or possibly contributing mechanism of increased fibrinolysis in cardiac arrest may be thrombin-related plasmin activation following blood exposure to interstitial tissue factor through hypoxic endothelial damage. Along these lines, Adrie et al (5) found a significant association between thrombin levels and organ dysfunction after successful resuscitation. Thrombin may also contribute to delayed t-PA (23) and/or urokinase-type plasminogen activator (24) release from endothelial cells.

In our study, patients’ prothrombin fragments were markedly elevated in both, patients with and without increased fibrinolysis, suggesting early thrombin generation in cardiac arrest. Yet, although thrombin-mediated coagulopathy likely becomes important hours or days after successful resuscitation, possibly as part of the postcardiac arrest syndrome, its contribution to increased fibrinolysis detected by thrombelastometry very early after successful resuscitation is questionable. Furthermore, in tissue factor/thrombin-driven coagulopathy, we would expect substantial consumption of clotting factors and platelets resulting in what is referred as DIC with fibrinolytic phenotype (25).

Consumptive coagulopathy, however, was very rare in our study patients, and the rate of overt DIC at admission did not differ between patients with and those without increased fibrinolysis. The low prevalence of overt DIC in our study patients, however, is in striking contrast to data from a retrospective Asian study, which reported overt DIC rates of 33% in resuscitated cardiac arrest with greater than 90% hospital mortality (26). As we analyzed a highly preselected subset of patients, the results of the two studies are difficult to compare. Yet, it would be of interest whether these differences result from different resuscitation policies (including termination-of-care rules) and whether overt DIC simply represents a premortem sign.

Another possible contributor to the impairment of coagulation may be hypothermia, but, however, a substantial impact on increased fibrinolysis in our patients seems unlikely. Median body temperature at admission was above 35°C, which is in agreement with recent literature (27), and did not differ significantly between patients with and without ML greater than or equal to 20%. Furthermore, previous studies did not report patterns of increased fibrinolysis under intentional hypothermia (28–31).

We were further interested in whether exogenous epinephrine may contribute to the extent of increased fibrinolysis in cardiac arrest. Catecholamines are known to promote release of t-PA from the endothelium (32), and the amount of epinephrine given during resuscitation has previously been linked to poor outcome regardless of the length of resuscitation (33). In the current study, there was an insignificant trend of higher cumulative epinephrine doses in patients who presented with ML greater than or equal to 20%. In regression analysis, however, the dosage of epinephrine administered had no effect on mortality after adjustment for ML greater than or equal to 20, which may suggest no causative relationship. Whether exogenous epinephrine directly contributes to fibrinolysis or simply reflects longer resuscitation efforts, and thus prolonged preceding hypoperfusion remains speculative and might be answered in a further study. Furthermore, yet, no data are available on the interaction between endogenous catecholamine levels and fibrinolysis in cardiac arrest. Our own recent data derived from a subset of 1,188 cardiac arrest patients underline the outcome-predictive role of sympathoadrenergic activation following resuscitation indicated by both, increased short- and long-term mortality in cardiac arrest along with the amount of immature peripheral neutrophils at admission (34). It is unclear whether this simply reflects the gradual extent of stress to the body following resuscitation or whether endogenous catecholamines may have a role in subsequent alterations of the coagulation and fibrinolytic system, as it has been described in trauma and septic patients (35, 36). In addition to increased fibrinolysis caused by stress-mediated sympathoadrenergic catecholamine release, neutrophil elastase has been linked as trigger for fibrinolysis in trauma (25). However, to date, no comparable data are available on the contribution of neutrophil elastase to promote fibrinolysis in cardiac arrest.

Some limitations need to be considered while interpreting the results. The current prospective observational study analyzed a strictly preselected cohort of OHCA patients including only those with presumed cardiac cause of cardiac arrest, who had achieved ROSC at admission, and were subjected to targeted temperature management. The possibly associated selection bias has to be considered, and study results need to be interpreted with appropriate caution.

Furthermore, it has to be mentioned that there is currently no widely accepted “gold standard” assay available for detection of systemic fibrinolysis. A possible risk of bias relating to the lack of a reference standard must be taken into account. Future studies determining thrombelastometry thresholds for increased fibrinolysis in OHCA need to validate their findings against standardized methods for detection of fibrinolysis, which may become available in the future (37). Further investigations also need to confirm the predictive performance of a greater than or equal to 20% ML cutoff found in this study.

## CONCLUSIONS

Increased fibrinolysis at admission may be interpreted as cumulative surrogate marker for hypoperfusion and hypoxia, that is, the duration of no-flow time and resuscitation quality. The current study provides a predictive cutoff value for a readily available bedside marker with 100% positive predictive value for poor outcome, which could be of interest for both treating physicians and relatives.

## ACKNOWLEDGMENT

We thank Gerhard Ruzicka, Karin Petroczi, and Christa Drucker for their valuable support with thrombelastometry and laboratory analysis.

## Supplementary Material

## References

[1] Theusinger OM Wanner GA Emmert MY Hyperfibrinolysis diagnosed by rotational thromboelastometry (ROTEM) is associated with higher mortality in patients with severe trauma. Anesth Analg 2011; 113:1003 1012 21918164 10.1213/ANE.0b013e31822e183f

[2] Ostrowski SR Windelov NA Ibsen M Consecutive thrombelastography clot strength profiles in patients with severe sepsis and their association with 28-day mortality: A prospective study. J Crit Care 2013; 28:317.e1—e11 10.1016/j.jcrc.2012.09.003 23159146

[3] Schöchl H Frietsch T Pavelka M Hyperfibrinolysis after major trauma: Differential diagnosis of lysis patterns and prognostic value of thrombelastometry. J Trauma 2009; 67:125 131 19590321 10.1097/TA.0b013e31818b2483

[4] Cotton BA Harvin JA Kostousouv V Hyperfibrinolysis at admission is an uncommon but highly lethal event associated with shock and prehospital fluid administration. J Trauma Acute Care Surg 2012; 73:365 370 22846941 10.1097/TA.0b013e31825c1234

[5] Adrie C Monchi M Laurent I Coagulopathy after successful cardiopulmonary resuscitation following cardiac arrest: Implication of the protein C anticoagulant pathway. J Am Coll Cardiol 2005; 46:21 28 15992630 10.1016/j.jacc.2005.03.046

[6] Schwameis M Schober A Schörgenhofer C Asphyxia by drowning induces massive bleeding due to hyperfibrinolytic disseminated intravascular coagulation. Crit Care Med 2015; 43:2394 2402 26327200 10.1097/CCM.0000000000001273 PMC4603369

[7] Viersen VA Greuters S Korfage AR Hyperfibrinolysis in out of hospital cardiac arrest is associated with markers of hypoperfusion. Resuscitation 2012; 83:1451 1455 22634432 10.1016/j.resuscitation.2012.05.008

[8] Deng Y He L Yang J Serum D-dimer as an indicator of immediate mortality in patients with in-hospital cardiac arrest. Thromb Res 2016; 143:161 165 26973153 10.1016/j.thromres.2016.03.001

[9] Szymanski FM Karpinski G Filipiak KJ Usefulness of the D-dimer concentration as a predictor of mortality in patients with out-of-hospital cardiac arrest. Am J Cardiol 2013; 112:467 471 23683952 10.1016/j.amjcard.2013.03.057

[10] Schöchl H Cadamuro J Seidl S Hyperfibrinolysis is common in out-of-hospital cardiac arrest: Results from a prospective observational thromboelastometry study. Resuscitation 2013; 84:454 459 22922072 10.1016/j.resuscitation.2012.08.318

[11] Testori C Sterz F Behringer W Surface cooling for induction of mild hypothermia in conscious healthy volunteers - a feasibility trial. Crit Care 2011; 15:R248 22018242 10.1186/cc10506 PMC3334799

[12] Jacobs I Nadkarni V Bahr J; International Liaison Committee on Resuscitation; American Heart Association; European Resuscitation Council; Australian Resuscitation Council; New Zealand Resuscitation Council; Heart and Stroke Foundation of Canada; InterAmerican Heart Foundation; Resuscitation Councils of Southern Africa; ILCOR Task Force on Cardiac Arrest and Cardiopulmonary Resuscitation Outcomes: Cardiac arrest and cardiopulmonary resuscitation outcome reports: Update and simplification of the Utstein templates for resuscitation registries: A statement for healthcare professionals from a task force of the International Liaison Committee on Resuscitation (American Heart Association, European Resuscitation Council, Australian Resuscitation Council, New Zealand Resuscitation Council, Heart and Stroke Foundation of Canada, InterAmerican Heart Foundation, Resuscitation Councils of Southern Africa). Circulation 2004; 110:3385 3397 15557386 10.1161/01.CIR.0000147236.85306.15

[13] Edgren E Hedstrand U Kelsey S Assessment of neurological prognosis in comatose survivors of cardiac arrest. BRCT I Study Group. Lancet 1994; 343:1055 1059 7909098 10.1016/s0140-6736(94)90179-1

[14] Taylor FB Jr Toh CH Hoots WK; Scientific Subcommittee on Disseminated Intravascular Coagulation (DIC) of the International Society on Thrombosis and Haemostasis (ISTH): Towards definition, clinical and laboratory criteria, and a scoring system for disseminated intravascular coagulation. Thromb Haemost 2001; 86:1327 1330 11816725

[15] Buderer NM Statistical methodology: I. Incorporating the prevalence of disease into the sample size calculation for sensitivity and specificity. Acad Emerg Med 1996; 3:895 900 8870764 10.1111/j.1553-2712.1996.tb03538.x

[16] Spiel AO Mayr FB Firbas C Validation of rotation thrombelastography in a model of systemic activation of fibrinolysis and coagulation in humans. J Thromb Haemost 2006; 4:411 416 16420574 10.1111/j.1538-7836.2006.01715.x

[17] Leitner JM Firbas C Mayr FB Recombinant human antithrombin inhibits thrombin formation and interleukin 6 release in human endotoxemia. Clin Pharmacol Ther 2006; 79:23 34 16413239 10.1016/j.clpt.2005.10.003

[18] Jilma-Stohlawetz P Fritsche-Polanz S Quehenberger P Evaluation of between-, within- and day-to-day variation of coagulation measured by rotational thrombelastometry (ROTEM). Scand J Clin Lab Invest 2017; 77:651 657 29081243 10.1080/00365513.2017.1394487

[19] Gando S Kameue T Nanzaki S Massive fibrin formation with consecutive impairment of fibrinolysis in patients with out-of-hospital cardiac arrest. Thromb Haemost 1997; 77:278 282 9157581

[20] Chandler WL Alessi MC Aillaud MF Clearance of tissue plasminogen activator (TPA) and TPA/plasminogen activator inhibitor type 1 (PAI-1) complex: Relationship to elevated TPA antigen in patients with high PAI-1 activity levels. Circulation 1997; 96:761 768 9264480 10.1161/01.cir.96.3.761

[21] Pecori Giraldi F Ambrogio AG Fatti LM Von Willebrand factor and fibrinolytic parameters during the desmopressin test in patients with Cushing’s disease. Br J Clin Pharmacol 2011; 71:132 136 21143510 10.1111/j.1365-2125.2010.03812.x PMC3018035

[22] Pinsky DJ Liao H Lawson CA Coordinated induction of plasminogen activator inhibitor-1 (PAI-1) and inhibition of plasminogen activator gene expression by hypoxia promotes pulmonary vascular fibrin deposition. J Clin Invest 1998; 102:919 928 9727060 10.1172/JCI307 PMC508957

[23] Levin EG Marzec U Anderson J Thrombin stimulates tissue plasminogen activator release from cultured human endothelial cells. J Clin Invest 1984; 74:1988 1995 6542570 10.1172/JCI111620 PMC425386

[24] Miles LA Levin EG Plescia J Plasminogen receptors, urokinase receptors, and their modulation on human endothelial cells. Blood 1988; 72:628 635 2840987

[25] Hayakawa M Sawamura A Gando S Disseminated intravascular coagulation at an early phase of trauma is associated with consumption coagulopathy and excessive fibrinolysis both by plasmin and neutrophil elastase. Surgery 2011; 149:221 230 20655560 10.1016/j.surg.2010.06.010

[26] Kim J Kim K Lee JH Prognostic implication of initial coagulopathy in out-of-hospital cardiac arrest. Resuscitation 2013; 84:48 53 22975022 10.1016/j.resuscitation.2012.09.003

[27] Nielsen N Wetterslev J Cronberg T; TTM Trial Investigators: Targeted temperature management at 33°C versus 36°C after cardiac arrest. N Engl J Med 2013; 369:2197 2206 24237006 10.1056/NEJMoa1310519

[28] Dirkmann D Hanke AA Görlinger K Hypothermia and acidosis synergistically impair coagulation in human whole blood. Anesth Analg 2008; 106:1627 1632 18499589 10.1213/ane.0b013e31817340ad

[29] Dirkmann D Radü-Berlemann J Görlinger K Recombinant tissue-type plasminogen activator-evoked hyperfibrinolysis is enhanced by acidosis and inhibited by hypothermia but still can be blocked by tranexamic acid. J Trauma Acute Care Surg 2013; 74:482 488 23354242 10.1097/TA.0b013e318280dec1

[30] Ruzicka J Stengl M Bolek L Hypothermic anticoagulation: Testing individual responses to graded severe hypothermia with thromboelastography. Blood Coagul Fibrinolysis 2012; 23:285 289 22356838 10.1097/MBC.0b013e328351885a

[31] Rundgren M Engström M A thromboelastometric evaluation of the effects of hypothermia on the coagulation system. Anesth Analg 2008; 107:1465 1468 18931200 10.1213/ane.0b013e31817ee955

[32] von Känel R Dimsdale JE Effects of sympathetic activation by adrenergic infusions on hemostasis in vivo. Eur J Haematol 2000; 65:357 369 11168493 10.1034/j.1600-0609.2000.065006357.x

[33] Dumas F Bougouin W Geri G Is epinephrine during cardiac arrest associated with worse outcomes in resuscitated patients? J Am Coll Cardiol 2014; 64:2360 2367 25465423 10.1016/j.jacc.2014.09.036

[34] Weiser C Schwameis M Sterz F Mortality in patients resuscitated from out-of-hospital cardiac arrest based on automated blood cell count and neutrophil lymphocyte ratio at admission. Resuscitation 2017; 116:49 55 28476480 10.1016/j.resuscitation.2017.05.006

[35] Ostrowski SR Henriksen HH Stensballe J Sympathoadrenal activation and endotheliopathy are drivers of hypocoagulability and hyperfibrinolysis in trauma: A prospective observational study of 404 severely injured patients. J Trauma Acute Care Surg 2017; 82:293 301 27779595 10.1097/TA.0000000000001304

[36] Johansson PI Haase N Perner A Association between sympathoadrenal activation, fibrinolysis, and endothelial damage in septic patients: A prospective study. J Crit Care 2014; 29:327 333 24581948 10.1016/j.jcrc.2013.10.028

[37] Longstaff C Measuring fibrinolysis: From research to routine diagnostic assays. J Thromb Haemost 2018; 16:652 662 29363269 10.1111/jth.13957 PMC5947570

