# Supplementary material for: Increased Fibrinolysis as a Specific Marker of Poor Outcome After Cardiac Arrest
Source: Crit Care Med. 2018 Sep 14;46(10):e995–e1001. doi: 10.1097/CCM.0000000000003352 (PMC6147086; doi:10.1097/CCM.0000000000003352)
Supplement: Supplementary file 1 [file ccm-46-0e995-s001.docx]

**Supplement**

**Supplemental Table 1.** ISTH DIC Score

| **Laboratory test (reference range)** | **Result** | **Points** |
| --- | --- | --- |
| Fibrinogen (200-400mg/dL) | <100 | 1 |
|  | >100 | 0 |
| D-Dimer (<0.5μg/mL) | <0.4 | 0 |
|  | 0.4-4 | 2 |
|  | >4 | 3 |
| Prothrombin time (>70%) | >70 | 0 |
|  | 40-70 | 1 |
|  | <40 | 2 |
| Platelet count (150-350x10^9^/L) | >100 | 0 |
|  | 100-50 | 1 |
|  | <50 | 2 |

Legend: A score ≥5 is compatible with overt DIC.

The ISTH DIC scoring-system (1-3)* proposes a validated diagnostic algorithm based on four coagulation parameters using a 8-point-scale from 0 to 8: fibrinogen (200-400mg/dL; <100mg/dL=1), d-dimer (<0.4μg/mL=0, 0.4-4μg/mL=2, >4μg/mL=3), prothrombin time (>70%=0, 40-70%=1, <40%=2) and platelets (150-350x10^9^/L; >100x10^9^/L=0, 100-50x10^9^/L=1, <50x10^9^/L=2).

*References:

1. Taylor FB, Jr., Toh CH, Hoots WK, et al. Towards definition, clinical and laboratory criteria, and a scoring system for disseminated intravascular coagulation. Thrombosis and haemostasis 2001; 86:1327-30.

2. Bakhtiari K, Meijers JC, de Jonge E, Levi M. Prospective validation of the International Society of Thrombosis and Haemostasis scoring system for disseminated intravascular coagulation. Critical care medicine 2004; 32:2416-21.

3. Toh CH, Hoots WK, ISTH SSCoDICot. The scoring system of the Scientific and Standardisation Committee on Disseminated Intravascular Coagulation of the International Society on Thrombosis and Haemostasis: a 5-year overview. *Journal of thrombosis and haemostasis : JTH*. 2007; **5**: 604-6. 10.1111/j.1538-7836.2007.02313.x.

**Supplemental Table 2.** Prevalence of ML ≥20% at different time points post admission: at target temperature (TT, 33±1°C), at 12 hours, at 24 hours and after rewarming

|  | | OUTCOME | | | |
| --- | --- | --- | --- | --- | --- |
|  |  | good (CPC1-2) | | poor (CPC3-5) | |
|  |  | Number | Percentage | Number | Percentage |
|  | ML at TT<20 | 34 | 100,0% | 40 | 100,0% |
|  | ML at TT≥20 | 0 | 0,0% | 0 | 0,0% |
|  | ML at 12h<20 | 35 | 100,0% | 39 | 100,0% |
|  | ML at 12h≥20 | 0 | 0,0% | 0 | 0,0% |
|  | ML at 24h<20 | 35 | 100,0% | 36 | 97,3% |
|  | ML at 24h≥20 | 0 | 0,0% | 1 | 2,7% |
|  | ML at RW<20 | 34 | 100,0% | 33 | 100,0% |
|  | ML at RW≥20 | 0 | 0,0% | 0 | 0,0% |

ML, maximum lysis; RW, rewarming; TT, target temperature

**Supplemental Table 3.** Tissue-type plasminogen activator (t-PA) antigen levels from admission to rewarming, stratified according to ML 20% cut-off at admission

| ML_T0 | | tPA_0h | | | tPA_12h | | | tPA_24h | | | tPA_RW_ | | | |
| --- | --- | --- | --- | --- | --- | --- | --- | --- | --- | --- | --- | --- | --- | --- |
|  |  | Median | Percentile 25 | Percentile 75 | Median | Percentile 25 | Percentile 75 | Median | Percentile 25 | Percentile 75 | Median | Percentile 25 | Percentile 75 |  |
|  | **ML_T0 <20** | **29,00** | 17,10 | 48,50 | **35,80** | 23,50 | 51,30 | **32,00** | 21,10 | 43,30 | **26,90** | 16,00 | 44,30 |  |
|  | **ML_T0 >=20** | **51,50** | 25,90 | 78,70 | **33,50** | 23,60 | 49,40 | **27,90** | 22,30 | 40,60 | **25,80** | 20,60 | 34,40 |  |

ML_T0, Maximum lysis at admission; tPA, tissue-type plasminogen activator; RW, rewarming

**Supplemental Table 4.** Exact logistic regression

**MAXIMUM LYSIS ≥20**

Exact logistic regression Number of obs = 78

Model score = 18.39344

Pr >= score = 0.0000

---------------------------------------------------------------------------

outcome1po~d | Odds Ratio Suff. 2*Pr(Suff.) [95% Conf. Interval]

-------------+-------------------------------------------------------------

mlt0_grg20 32.60495* 17 0.0000 5.187938 +Inf

---------------------------------------------------------------------------

(*) median unbiased estimates (MUE)

. exlogistic outcome1poor0good mlt020120020 tert_age, memory(2g) nolog

note: CMLE estimate for mlt020120020 is +inf; computing MUE

**AGE**

Exact logistic regression Number of obs = 78

Model score = 22.12829

Pr >= score = 0.0000

---------------------------------------------------------------------------

outcome1po~d | Odds Ratio Suff. 2*Pr(Suff.) [95% Conf. Interval]

-------------+-------------------------------------------------------------

mlt0_grg20 | 29.45064* 17 0.0000 4.595827 +Inf

tert_age | 2.062067 53 0.0425 1.022295 4.383727

---------------------------------------------------------------------------

(*) median unbiased estimates (MUE)

. exlogistic outcome1poor0good mlt020120020 tert_supra, memory(2g) nolog

note: CMLE estimate for mlt020120020 is +inf; computing MUE

**EPINEPHRINE DOSE**

Exact logistic regression Number of obs = 76

Model score = 22.59132

Pr >= score = 0.0000

---------------------------------------------------------------------------

outcome1po~d | Odds Ratio Suff. 2*Pr(Suff.) [95% Conf. Interval]

-------------+-------------------------------------------------------------

mlt0_grg20 | 29.19097* 17 0.0000 4.542853 +Inf

tert_supra | 2.447561 57 0.0354 1.054549 6.240308

---------------------------------------------------------------------------

(*) median unbiased estimates (MUE)

. exlogistic outcome1poor0good mlt020120020 tert_dd, memory(2g) nolog

note: CMLE estimate for mlt020120020 is +inf; computing MUE

**D-DIMER**

Exact logistic regression Number of obs = 78

Model score = 27.28739

Pr >= score = 0.0000

---------------------------------------------------------------------------

outcome1po~d | Odds Ratio Suff. 2*Pr(Suff.) [95% Conf. Interval]

-------------+-------------------------------------------------------------

mlt0_grg20 | 23.64068* 17 0.0003 3.500902 +Inf

_d-dimer | 3.253603 57 0.0013 1.512612 7.734495

---------------------------------------------------------------------------

(*) median unbiased estimates (MUE)

. exlogistic outcome1poor0good mlt020120020 tert_ph, memory(2g) nolog

note: CMLE estimate for mlt020120020 is +inf; computing MUE

**PH**

Exact logistic regression Number of obs = 76

Model score = 29.19827

Pr >= score = 0.0000

---------------------------------------------------------------------------

outcome1po~d | Odds Ratio Suff. 2*Pr(Suff.) [95% Conf. Interval]

-------------+-------------------------------------------------------------

mlt0_grg20 | 30.37619* 17 0.0001 4.347984 +Inf

ph | .2405486 27 0.0003 .0900247 .5553192

---------------------------------------------------------------------------

(*) median unbiased estimates (MUE)

. exlogistic outcome1poor0good mlt020120020 tert_lact, memory(2g) nolog

note: CMLE estimate for mlt020120020 is +inf; computing MUE

**LACTATE**

Exact logistic regression Number of obs = 76

Model score = 25.88119

Pr >= score = 0.0000

---------------------------------------------------------------------------

outcome1po~d | Odds Ratio Suff. 2*Pr(Suff.) [95% Conf. Interval]

-------------+-------------------------------------------------------------

mlt0_grg20 | 31.97409* 17 0.0000 4.823866 +Inf

lactate | 2.821249 55 0.0032 1.368843 6.303404

---------------------------------------------------------------------------

(*) median unbiased estimates (MUE)

. exlogistic outcome1poor0good mlt020120020 tert_lowflow, memory(2g) nolog

note: CMLE estimate for mlt020120020 is +inf; computing MUE

**LOW FLOW TIME**

Exact logistic regression Number of obs = 78

Model score = 25.64101

Pr >= score = 0.0000

---------------------------------------------------------------------------

outcome1po~d | Odds Ratio Suff. 2*Pr(Suff.) [95% Conf. Interval]

-------------+-------------------------------------------------------------

mlt0_grg20 | 23.95812* 17 0.0003 3.526907 +Inf

_lowflow | 3.0372 57 0.0031 1.398437 7.268179

---------------------------------------------------------------------------

(*) median unbiased estimates (MUE)

**NO FLOW TIME**

Exact logistic regression Number of obs = 73

Model score = 19.00272

Pr >= score = 0.0000

---------------------------------------------------------------------------

pooroutcome | Odds Ratio Suff. 2*Pr(Suff.) [95% Conf. Interval]

-------------+-------------------------------------------------------------

mlt0_gr20 | 29.96375* 17 0.0000 4.702129 +Inf

**noflow** | 1.228006 50 0.2550 .881127 1.842335

---------------------------------------------------------------------------

(*) median unbiased estimates (MUE)

. exlogistic pooroutcome mlt0_gr20 male, memory(2g) nolog

note: CMLE estimate for mlt0_gr20 is +inf; computing MUE

**SEX**

Exact logistic regression Number of obs = 78

Model score = 18.65223

Pr >= score = 0.0000

---------------------------------------------------------------------------

pooroutcome | Odds Ratio Suff. 2*Pr(Suff.) [95% Conf. Interval]

-------------+-------------------------------------------------------------

mlt0_gr20 | 28.51553* 17 0.0000 4.517236 +Inf

**male** | 1.538636 12 0.7685 .3106902 7.642624

---------------------------------------------------------------------------

(*) median unbiased estimates (MUE)

. exlogistic pooroutcome mlt0_gr20 cpc_prior_bad, memory(2g) nolog

note: CMLE estimate for mlt0_gr20 is +inf; computing MUE

note: CMLE estimate for cpc_prior_~d is +inf; computing MUE

**CPC PRIOR TO CARDIAC ARREST**

Exact logistic regression Number of obs = 78

Model score = 21.25424

Pr >= score = 0.0000

---------------------------------------------------------------------------

pooroutcome | Odds Ratio Suff. 2*Pr(Suff.) [95% Conf. Interval]

-------------+-------------------------------------------------------------

mlt0_grg20 | 35.29032* 17 0.0000 5.597599 +Inf

**cpc_prior_~d** | 3.585665* 2 0.3279 .2736752 +Inf

---------------------------------------------------------------------------

(*) median unbiased estimates (MUE)

. exlogistic pooroutcome mlt0_gr20 home, memory(2g) nolog

note: CMLE estimate for mlt0_gr20 is +inf; computing MUE

**ARREST SITE**

Exact logistic regression Number of obs = 78

Model score = 18.39613

Pr >= score = 0.0001

---------------------------------------------------------------------------

pooroutcome | Odds Ratio Suff. 2*Pr(Suff.) [95% Conf. Interval]

-------------+-------------------------------------------------------------

mlt0_grg20 | 31.40646* 17 0.0000 5.030632 +Inf

**home** | .969794 21 1.0000 .3096672 3.043459

---------------------------------------------------------------------------

(*) median unbiased estimates (MUE)

. exlogistic pooroutcome mlt0_gr20 witnessed, memory(2g) nolog

note: CMLE estimate for mlt0_gr20 is +inf; computing MUE

**WITNESS STATUS**

Exact logistic regression Number of obs = 78

Model score = 19.65134

Pr >= score = 0.0000

---------------------------------------------------------------------------

pooroutcome | Odds Ratio Suff. 2*Pr(Suff.) [95% Conf. Interval]

-------------+-------------------------------------------------------------

mlt0_grg20 | 31.90985* 17 0.0000 5.042526 +Inf

**witnessed** | .4021657 32 0.3243 .0735157 1.945538

---------------------------------------------------------------------------

(*) median unbiased estimates (MUE)

. exlogistic pooroutcome mlt0_gr20 bystander, memory(2g) nolog

note: CMLE estimate for mlt0_gr20 is +inf; computing MUE

**BYSTANDER CPR**

Exact logistic regression Number of obs = 78

Model score = 18.66175

Pr >= score = 0.0000

---------------------------------------------------------------------------

pooroutcome | Odds Ratio Suff. 2*Pr(Suff.) [95% Conf. Interval]

-------------+-------------------------------------------------------------

mlt0_grg20 | 31.87677* 17 0.0000 5.084683 +Inf

**bystander** | .7124131 29 0.7522 .1984118 2.577457

---------------------------------------------------------------------------

(*) median unbiased estimates (MUE)

. exlogistic pooroutcome mlt0_gr20 nonshockable, memory(2g) nolog

note: CMLE estimate for mlt0_gr20 is +inf; computing MUE

**INITIAL RHYTHM**

Exact logistic regression Number of obs = 76

Model score = 22.29536

Pr >= score = 0.0000

---------------------------------------------------------------------------

pooroutcome | Odds Ratio Suff. 2*Pr(Suff.) [95% Conf. Interval]

-------------+-------------------------------------------------------------

mlt0_grg20 | 29.74196* 17 0.0000 4.573928 +Inf

**nonshockable** | 6.566611 13 0.0371 1.092369 71.52

---------------------------------------------------------------------------

(*) median unbiased estimates (MUE)

. exlogistic pooroutcome mlt0_gr20 sustrosc_onadmission, memory(2g) nolog

note: CMLE estimate for mlt0_gr20 is +inf; computing MUE

**SUSTAINED ROSC AT ADMISSION**

Exact logistic regression Number of obs = 78

Model score = 18.43275

Pr >= score = 0.0000

---------------------------------------------------------------------------

pooroutcome | Odds Ratio Suff. 2*Pr(Suff.) [95% Conf. Interval]

-------------+-------------------------------------------------------------

mlt0_grg20 | 30.57677* 17 0.0000 4.885397 +Inf

**sustrosc_o~n** | 1.404084 38 1.0000 .0693744 86.7114

---------------------------------------------------------------------------

(*) median unbiased estimates (MUE)

**Supplemental Figure 1.** Probability of survival to day 30 according to maximum fibrinolysis cut-off of ≥20% derived from receiver-operating-characteristic curve analysis. Dashed lines: 95% confidence interval at each time point plotted as confidence bands, ML, maximum lysis

**Supplemental Figure 2.** Graphical representation of thrombelastometric tests as temogram.

Rich-colored temogram of a patient with 100% maximum lysis and pale-colored temogram of a patient with 7% maximum lysis.


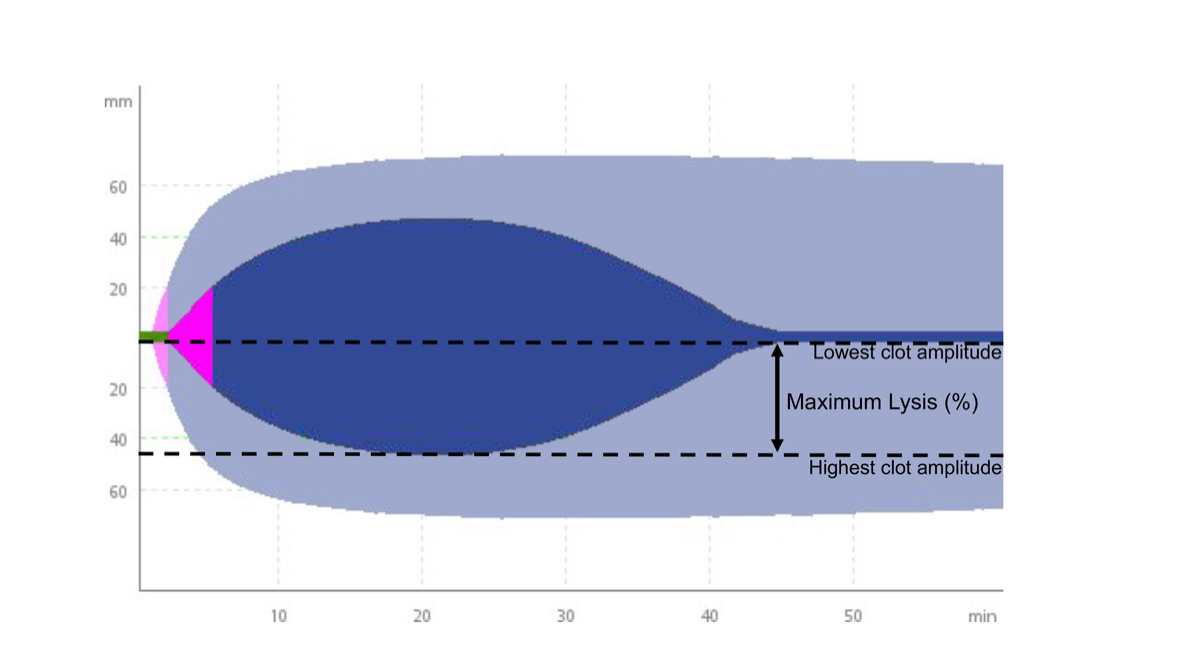


**Supplemental Figure 3.** Receiver-operating-characteristic curve of maximum lysis for prediction of poor neurologic outcome or death at day 30.

The optimal maximum lysis cut-off with 100% specificity was ≥ 20%. Corresponding sensitivity was 41%.

**Supplemental Figure 4.** Specificity (grey squares, %), sensitivity (black dots, %) and cumulative frequency distribution (light-colored and rich-colored red area, %; right y-axis) of potential predictor variables. The red vertical line marks the co-variable’s 100% specificity value for poor neurologic function or death. The rich-colored red area represents the percentage of patients that can be predicted with 100% specificity by applying the respective cut-off. These are 3% (age, no flow time and epinephrine dose), 5% (pH) and 7% (lactate) of all patients, respectively. Low-flow time values did not reach 100% specificity at all.
